# Self-Assembled Matrix by Umbilical Cord Stem Cells

**DOI:** 10.3390/jfb2030213

**Published:** 2011-09-01

**Authors:** Dimitrios Karamichos, Celeste B. Rich, Audrey E.K. Hutcheon, Ruiyi Ren, Biagio Saitta, Vickery Trinkaus-Randall, James D. Zieske

**Affiliations:** 1 Department of Ophthalmology, Schepens Eye Research Institute, Harvard Medical School, 20 Staniford Street, Boston MA 02114, USA; E-Mails: dimitris.karamichos@schepens.harvard.edu (D.K.); audrey.hutcheon@schepens.harvard.edu (A.E.K.H.); 2 Department of Biochemistry, Boston University School of Medicine, 80 E Concord Street, Boston, MA 02118, USA; E-Mails: cbrich@bu.edu (C.B.R.); renruiyi@bu.edu (R.R.); vickery@bu.edu (V.T.); 3 Department of Biomedical Sciences, Medical Genetics and Regenerative Medicine Institute, Cedars-Sinai Medical Center, 8727 W. Third Street, Metro Building, Suite 203, Los Angeles, CA 90048, USA; E-Mail: saittabiagio@gmail.com

**Keywords:** stem cells, extracellular matrix, cornea, glycosaminoglycans

## Abstract

Corneal integrity is critical for vision. Corneal wounds frequently heal with scarring that impairs vision. Recently, human umbilical cord mesenchymal stem cells (cord stem cells) have been investigated for tissue engineering and therapy due to their availability and differentiation potential. In this study, we used cord stem cells in a 3-dimensional (3D) stroma-like model to observe extracellular matrix organization, with human corneal fibroblasts acting as a control. For 4 weeks, the cells were stimulated with a stable Vitamin C (VitC) derivative ±TGF-β1. After 4 weeks, the mean thickness of the constructs was ∼30 μm; however, cord stem cell constructs had 50% less cells per unit volume, indicating the formation of a dense matrix. We found minimal change in decorin and lumican mRNA, and a significant increase in perlecan mRNA in the presence of TGF-β1. Keratocan on the other hand decreased with TGF-β1 in both cell lineages. With both cell types, the constructs possessed aligned collagen fibrils and associated glycosaminoglycans. Fibril diameters did not change with TGF-β1 stimulation or cell lineage; however, highly sulfated glycosaminoglycans associated with the collagen fibrils significantly increased with TGF-β1. Overall, we have shown that cord stem cells can secrete their own extracellular matrix and promote the deposition and sulfation of various proteoglycans. Furthermore, these cells are at least comparable to commonly used corneal fibroblasts and present an alternative for the 3D *in vitro* tissue engineered model.

## 1. Introduction

One of the main threats for the human eye following injury or disease is the development of a scar, which can compromise a person's vision and even lead to blindness. Treatment options are generally limited to corneal transplants, although several investigations have examined the potential use of biomaterials as a corneal substitute. Recently, studies have considered the possibility of stem cell transplantation. Stem cells have the potential to repair damaged organs and tissues, and they are also known for their unique intrinsic characteristics, which enable them to control cell replacement during homeostasis and tissue repair [1]. The characteristics of stem cells make them highly attractive for use in tissue engineering and artificial *in vitro* applications.

To date, the most commonly used source of adult mesenchymal stem cells (MSCs) is the bone marrow (BMSCs); however, MSCs are also found in adipose tissue, blood, dermis and fat. Researchers [2,3] have shown that BMSCs contribute to the regeneration of a variety of tissues, such as bone, cartilage, muscle, ligament, tendon, adipose and stroma [2,3]. Unfortunately, there are a few draw backs to BMSCs that are leading scientists to seek alternative stem cell sources: (1) BMSCs are found in low numbers in tissues, (2) Tissue and organ availability is limited and (3) BMSCs differentiation rate decreases over time [4]. In recent years, umbilical cord blood stem cells (cord stem cells) have been presented as an alternative to BMSCs. Cord stem cells are extracted from either cord blood or the Wharton's jelly of umbilical cords through enzyme digestion [5] and have a number of critical advantages over other MSCs: (1) the tissue is routinely discarded; therefore, the tissue is available for cord stem cell extraction; (2) the collection process of the tissue is non-invasive; and (3) there is no donor risk to an organ or tissue.

In the cornea, the three major cellular layers (epithelium, endothelium and stroma) may need restoration following injury. Currently, tissue engineered corneal epithelial cell sheets are used clinically in patients with total limbal stem cell deficiencies [6,7,8,9], and corneal endothelial cell sheets have, in fact, successfully restored corneal transparency in animal models [10,11,12,13]. However, none of the cell-based tissue engineered corneal stromal substitutes have been clinically feasible, although several kinds of tissue engineered corneal stroma have been reported [14]. Recently, our group has shown that human corneal fibroblasts retain the ability to deposit a complex extracellular matrix when stimulated with Vitamin C (VitC), and this ability is enhanced with the presence of specific growth factors, which drive the construct toward an *in vitro* stroma-like model. In this study, we investigated the ability of the cord stem cells to stratify and deposit an organized and functional matrix that is comparable to that deposited by corneal fibroblasts. Their ability to organize and align their own extracellular matrix is potentially crucial for their use as a corneal biomaterial.

The major proteins of the stromal matrix include the collagens and the proteoglycans with their associated glycosaminoglycan (GAG) chains. GAGs are extremely diverse due to the number of ways that they can be altered, including addition of sulfate moieties [15,16]. Chondroitin sulfate/dermatan sulfate side chains are associated with decorin and biglycan, while keratan side chains are associated with lumican [17,18]. In addition, heparan sulfate proteoglycans are not detected in the healthy uninjured cornea [19]. The knowledge of proteoglycan core regulation and GAG synthesis by two different cell lineages can be vital when developing corneal substitutes *in vitro*.

In our study, cord stem cells deposited collagen in an aligned configuration with a mean diameter similar to that of collagen in normal human stromas. The core proteoglycan, perlecan, showed an increase in mRNA in response to TGF-β1, along with the presence of sulfated GAGs. The development of such a model using cord stem cells may provide an alternative source of tissue for corneal transplantation.

## 2. Materials and Methods

### 2.1. Establishment of Umbilical Cord Mesenchymal Stem Cells

Umbilical cord mesenchymal stem cells (cord stem cells) were obtained from Dr. Biagio Saitta at Cedars-Sinai Medical Center/Regenerative Medicine Institute, Las Angeles, CA, and were isolated as previously reported [20]. Briefly, cells were harvested at birth from the placenta of full-term deliveries with the consent of the parents. This was done under the International Review Board-approved protocol to the New Jersey Cord Blood Bank, where Saitta previously worked. Blood was collected within 10 min of placenta removal and was drained into a plastic bag with 25 mL of citrate phosphate dextrose anticoagulant solution (Medsep Corporation; Covina, CA). Cells were processed as reported previously [20] and were used between passages 5–10. Detailed characterization and expression markers can be found in Markov *et al.*'s manuscript [20].

### 2.2. Fibroblast Assembled Extracellular Matrix

The cord stem cells were plated on transwell 6-well plates containing 24-mm polycarbonate membrane inserts with 0.4 μm pores (Costar; Charlotte, NC) at a density of 2 × 10^6^ cells in 1.5 mL EMEM. The seeding density was determined by a number of preliminary experiments where the amount of extracellular matrix deposited was the critical element. Constructs were allowed to grow for 4 weeks in either VitC media—EMEM with 10% FBS and 0.5 mM 2-O-α-D-glucopyranosyl-L-ascorbic acid (VitC: Wako Chemicals USA, Inc.; Richmond, V.A.)—only (control), or VitC media + 0.1 ng/mL TGF-β1 (TGF-β1 treated). Preliminary concentration-dependent experiments with TGF-β1 ruled out higher concentrations of the growth factor due to construct contraction. At week 4, samples of the resulting constructs were collected and processed for immunofluorescence microscopy, transmission electron microscopy (TEM) and Real Time RT-PCR.

### 2.3. Thickness

Constructs were fixed in 4% paraformaldehyde and processed for immunofluorescence, as previously described [21,22]. Constructs were stained with phalloidin-rhodamine (Invitrogen; Carlsbad, CA, USA), which binds and stains the f-actin filaments and allows for the visualization of all cells within the construct. Also, TOPRO-3 iodide (Invitrogen) was used to stain the cell nuclei. The constructs were imaged on a TCS-SP2 confocal microscope (Leica Microsystems; Bannockburn, I.L., UK). The total thickness of the constructs was calculated using the confocal z-series, where we measured from the very top cell to the very bottom one. To obtain a visual confirmation of this measurement, orthogonal sections of the same confocal z-series were created with the Image Pro Plus software.

### 2.4. Transmission Electron Microscopy

At 4 weeks, the constructs were fixed in ½ strength Karnovsky's fixative (2% paraformaldehyde, 2.5% glutaraldehyde in cacodylate buffer, pH 7.4) and processed, as previously described [23]. Briefly, samples were cut transversely to the plane of the construct, using a diamond knife ultramicrotome (LKB ultramicrotome; Bromma, Sweden). Sections (60–90 Å) were obtained, examined and photographed with a Philips 410 Transmission Electron Microscope (Philips Electronics N.V.; Eindhoven, The Netherlands) equipped with a digital camera.

### 2.5. Cell Numbers and Matrix Production

Using Image Pro Plus (v.7: Media Cybernetics; Bethesda, MD, USA), we quantified the total cell number per construct per condition, as well as, cell per unit volume. A minimum of 3 confocal z-series was used for each condition, and their average was plotted and analyzed.

### 2.6. Real Time RT-PCR

Total RNA was extracted using the RNeasy kit (Qiagen; Valencia, CA, USA). Genomic DNA was removed by incubation with RNase-free DNase I (M0303S: New England BioLabs; Ipswich, MA, UK) in the presence of RNase inhibitor. The RNA was annealed with random hexamer and oligo-dt primers, and first strand synthesis was carried out with MuLV reverse transcriptase. Negative controls were performed without reverse transcriptase. Real-Time PCR was performed on an Applied Biosystems 7300 Real-Time PCR system (Foster City, CA, USA) using ABI TaqMan human gene expression assays for perlecan (Hs01078536.m1), keratocan (Hs00559942.m1), decorin (Hs00370384.m1), lumican (Hs00158940.m1), collagen 1A1 (Hs00164004.m1), collagen 5A1 (Hs00619088.m1), and the eukaryotic 18S rRNA endogenous control (4308329), which was used for normalization. The cycling parameters were as follows: 50 °C for 2 min, 95 °C for 10 min, 40 cycles of 95 °C for 15 s and 60 °C for 1 min. Calculations were carried out using the δδCt method of relative quantitation. Results were expressed as the mean of relative mRNA levels ± SEM.

### 2.7. Cuprolinic Blue Staining and GAG Side Chain Measurements

Cuprolinic blue is an electron-dense reagent which has been shown to identify sulfated GAGs and to demonstrate the association of sulfated GAGs with matrix molecules, such as collagen [24]. The length of cuprolinic blue stained filaments indicates the extent of their sulfation. Briefly, the samples were stained with 0.2% Cuprolinic blue (in buffer containing 0.3 M MgCl_2_) for 24 hours, dehydrated and processed for electron microscopy [25,26,27].

One hundred cuprolinic blue stained GAG side chains were randomly chosen from each region—apical, middle and basal—of each construct, and their length were measured using ImageJ v.1.5 [28]. Results were analyzed for significance (p < 0.05) and plotted (Prism v.5.0: GraphPad Software, Inc.; La Jolla, CA, USA) [27].

### 2.8. Collagen Fibril Diameter Measurements

Fibril diameters for each condition were measured and compared using Photoshop (v.10.0.1). The scale bar for the TEM was used to calibrate the measurements. At least ten randomly chosen electron micrographs were used for each condition and a total of more than 100 fibrils were measured. Results were plotted and analyzed for significance (p < 0.05).

### 2.9. Statistical Analysis

All experiments were repeated at least three times and data was analyzed for significant variations (p < 0.05) using the Student's t-test and Dunnett's Multiple Comparison test.

## 3. Results

### 3.1. Construct Characterization

In our previous work [21,22,29], we have shown that corneal fibroblasts have the ability to secrete and lay down a fibrous collagen extracellular matrix in the presence of derivatized VitC. We have demonstrated the effect of TGF-β1, -β2 and -β3 in these cultures [22]. In the current study, we examined the ability of cord stem cells to synthesize a matrix in the presence of VitC ± TGF-β1. The development of an extracellular matrix *in vitro* would be useful for many applications in stem cell tissue engineering.

After 4 weeks, cord stem cells cultured in the presence of VitC resulted in a construct approximately 35 um thick, which is similar to constructs synthesized by human corneal fibroblasts under the same conditions (Figure 1.1 and 1.2A, C) [21,22]. Surprisingly, when TGF-β1 was added to the cord stem cell cultures, no significant increase was found in the construct thickness (Figure 1.1 and 1.2D). This is in contrast to constructs synthesized by human corneal fibroblasts, where the thickness increased 2–3 fold (p < 0.0001; Figure 1.1 and 1.2A, B). Results were confirmed and shown in Figure 1.2 using orthogonal sections created with Image Pro Plus software. This data suggests that a differential path of expression may exist between the two cell types.

**Figure 1:**
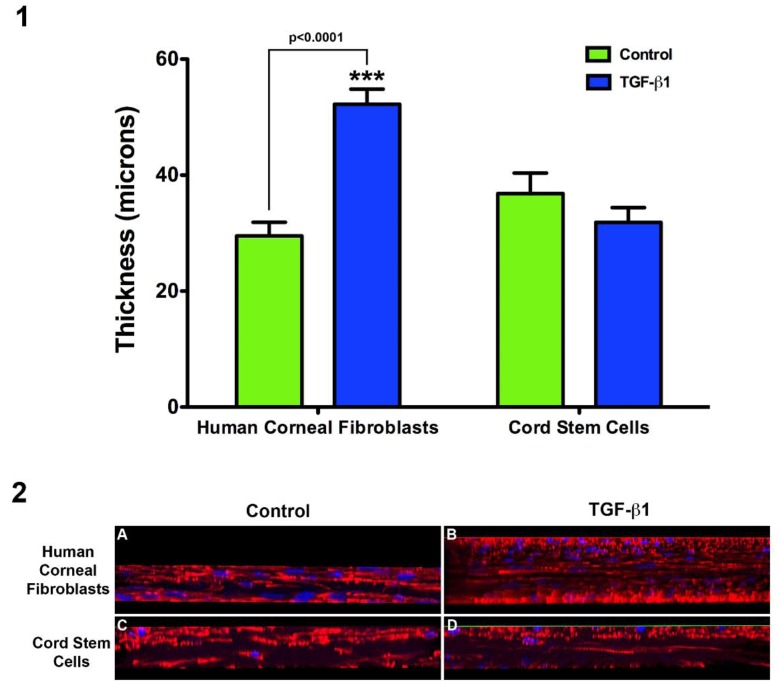
Total construct thickness for Control and TGF-β1 treated human corneal fibroblasts and cord stem cells. (**1**) Graphical representation of construct thickness. Note that human corneal fibroblast constructs' thickness was significantly increased when stimulated with TGF-β1 (p < 0.0001), whereas cord stem cells showed no significant changes between Control and TGF-β1 treatment; (**2**) Orthogonal view of constructs after 3D reconstruction of images captured by confocal microscopy with a 40× objective; (**2A** and **B**) Human corneal fibroblast constructs, Control and TGF-β1, respectively; and (**2C** and **D**) Cord stem cells, Control and TGF-β1, respectively. Red = Phalloidin, Blue = TOPRO-3.

### 3.2. Ultrastructural Studies

**Figure 2:**
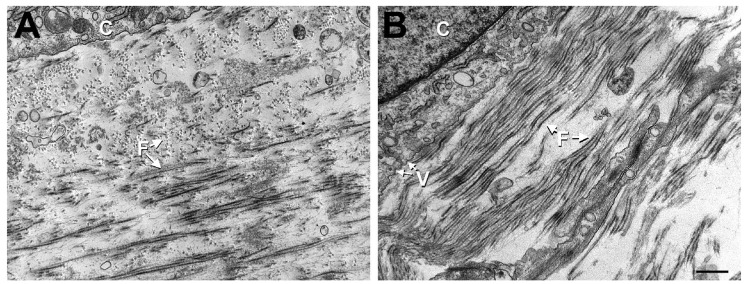
TEM (31,000×) showing cell-matrix interaction of cord stem cells in two different conditions: (**A**) Control and (**B**) TGF-β1 treated. Note: Fibril (F) alignment alternated direction in control and to a much lesser extent in TGF-β1 treated; however, TGF-β1 treatment (B) showed higher fibril density and more alignment than Control (A). C = cord stem cells. An example of a cell with vesicles (V) is shown in both A and B, indicating the presence of myofibroblasts in both conditions with more of these cells present in B. Bar = 0.5 microns.

Cell-matrix interactions revealed similarities between the two cell lineages. Cord stem cells assembled aligned collagen that alternated directions in Controls (Figure 2A). When stimulated with TGF-β1 (Figure 2B), the number of alternating layers appeared to decrease resulting in a higher degree of alignment. This observation was similar to what was seen previously with corneal fibroblasts [21]. Also, in the cord stem cell constructs, cells were elongated and very similar in morphology to human corneal fibroblasts (data not shown), with the addition of TGF-β1, these cells resulted in more aligned actin-like filaments, indicative of myofibroblasts (data not shown). Vesicles were also seen under both conditions (Figure 2); however, they were more abundant when constructs were treated with TGF-β1 (Figure 2B). Vesicles were recently identified by us and reported as a potential myofibroblast marker [22].

### 3.3. Cell Numbers and Extracellular Matrix Production

**Figure 3:**
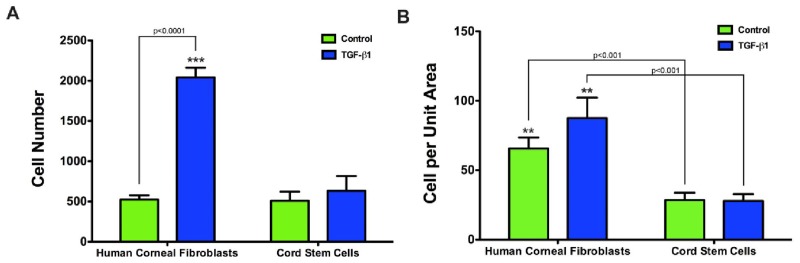
Graphs of the total number of cells present (A), and total number of cells per unit volume (mm^2^) (B), within the two types of constructs. (**A**) In control constructs for both human corneal fibroblasts and cord stem cells, identical number of cells was apparent; however, upon the addition of TGF-β1, the number of cells in the human corneal fibroblast constructs were significantly upregulated (p < 0.0001), whereas the cord stem cell number remained relatively unchanged; (**B**) Human corneal fibroblast constructs had more cells per unit volume compared to cord stem cells (p < 0.001) in both control and TGF-β1 treatment. Human corneal fibroblast cell numbers per unit volume increased slightly with TGF-β1 treatment, where cord stem cells remained unchanged.

For each sample, the cell number and the resulting cell number per unit volume were calculated using Image Pro Plus. When cells were stimulated with TGF-β1, human corneal fibroblast constructs had a higher total number of cells (∼3 fold) compared to cord stem cell constructs (p < 0.0001; Figure 3A). However, there was no difference in the control groups for either lineage. Taking into account that the control construct thickness for both cell lineages was approximately equal, and only the thickness of the human corneal fibroblast construct increased with the addition of TGF-β1 (Figure 1), we calculated the cell number per unit volume for all conditions. The data showed that there was no difference in the cell number per unit volume between control and TGF-β1 stimulated cord stem cell constructs (Figure 3B). However, human corneal fibroblast constructs increased in cell number per unit volume upon TGF-β1 stimulation (Figure 3B). Human corneal fibroblasts showed significantly higher numbers of cells per unit volume, both in Controls and TGF-β1 when compared to cord stem cells (p < 0.001). Overall our data indicates that the cord stem cells assembled a more extensive matrix per cell than the human corneal fibroblasts.

### 3.4. Collagen

**Figure 4:**
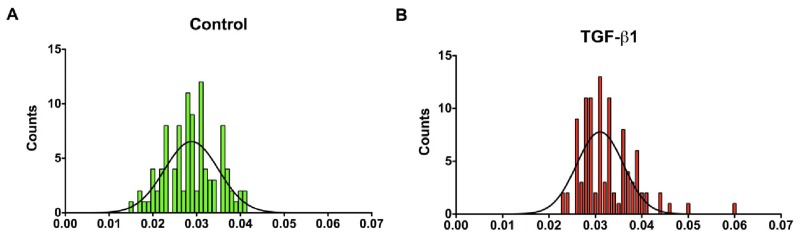
Histograms demonstrating the distribution of cord stem cell construct fibril diameters under two conditions: (**A**) Control and (**B**) TGF-β1 treated. (**A**) Control fibril diameters ranged from 15 to 42 nm with a mean diameter at 28 nm; (**B**) TGF-β1 showed a range from 23 to 60 nm with mean size of 32 nm. This shift of fibril diameters was significantly different (p < 0.001).

In our previous work with human corneal fibroblasts [22], we found that the Control and TGF-β1 treated construct fibril diameter means were 27 and 32 nm, respectively, which was similar to that seen in the mature human corneal stroma, 30–35 nm (hydrated type I/V collagen). In the present study with the cord stem cells, the fibril diameters in the Control constructs (Figure 4A) had a range of 15–42 nm, with a mean diameter of 28 nm. In response to TGF-β1 (Figure 4B), the mean fibril diameter was 32 nm, with a range of 23–60 nm. Thus, TGF-β1 slightly, but significantly (p < 0.001), increased the mean collagen fibril diameter of the cord stem cells. The data indicates that the fibril diameters of the cord stem cells were similar to those seen in the human corneal fibroblast constructs and the mature human corneal stroma. Of note, following injury in human corneal stroma, the fibril diameter range was found to increase (15–65 nm) [30].

**Figure 5:**
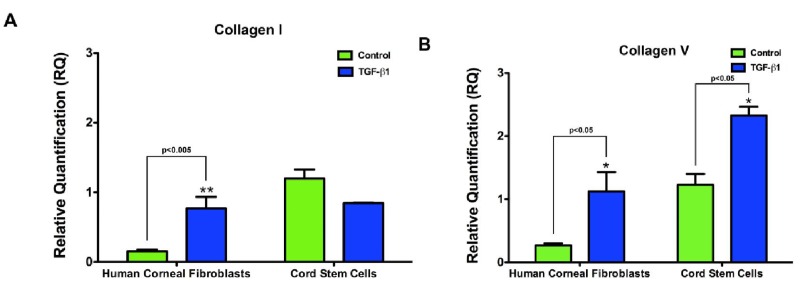
Relative quantification of Collagen Type I and V mRNA after Real Time RT-PCR. (**A**) Collagen I mRNA was significantly upregulated (p < 0.005) in the human corneal fibroblast constructs stimulated with TGF-β1; and (**B**) Collagen V mRNA expression was significantly upregulated after TGF-β1 stimulation in both cell lineages (p < 0.05).

As Type I and V collagen are known to be the major stromal collagens, we examined their expression in the construct to observe if their expression was modulated by cord stem cells in the presence of TGF-β1 (Figure 5). Collagen I (Figure 5A) mRNA was only enhanced in the constructs synthesized by human corneal fibroblasts (p < 0.005). We found that TGF-β1 enhanced the Collagen V mRNA expression (Figure 5B) in both cell lineages (p < 0.05), indicating that the constructs both showed a response to changes in extracellular matrix independent of the cell/matrix density.

### 3.5. GAG Expression and Deposition

Proteoglycans are known to play an important role in extracellular matrix organization in development and during pathology. Previously, in human corneal fibroblast 3D construct, the expression of proteoglycan cores (lumican, decorin and keratocan) was detected. In addition, the presence of associated sulfated GAGs was examined throughout the depth of the construct [27]. Since the regulation of proteoglycans may be critical for the design of a cornea-like construct, one of the goals of this study was to examine the expression of proteoglycan transcripts in the cord stem cell constructs.

**Figure 6:**
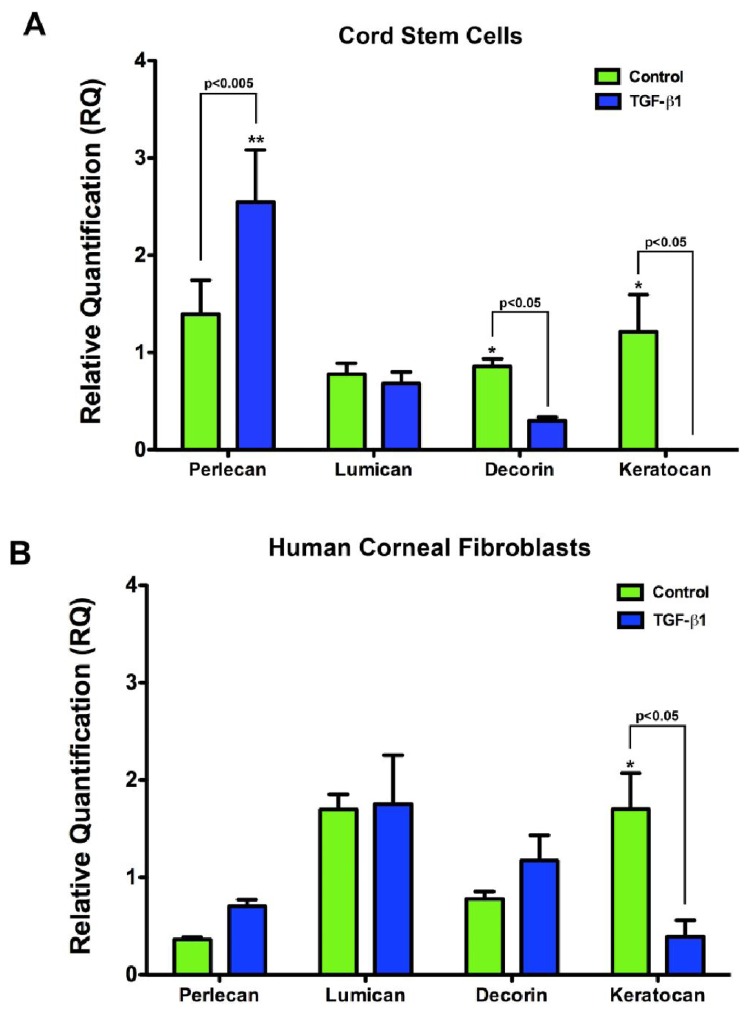
Real Time RT-PCR analysis of proteoglycans: lumican, keratocan, decorin, and perlecan. (**A**) In the cord stem cell constructs, perlecan was significantly upregulated (p < 0.005) with the addition of TGF-β1, and decorin and keratocan were significantly downregulated (p < 0.05); and (**B**) In the human corneal fibroblast constructs, keratocan was significantly downregulated (p < 0.05) with the addition of TGF-β1; whereas, the other proteoglycans remained relatively the same, with only slight increases.

The expression of proteoglycan core transcripts was performed using Real Time RT-PCR. In the cord stem cell constructs, there was a ∼2-fold decrease in decorin and keratocan mRNA (p < 0.05) when the cultures were incubated in the presence of TGF-β1 (Figure 6A). While there was no detectable change in lumican mRNA, there was a significant increase (p < 0.05) in perlecan mRNA (Figure 6A). This regulation was similar to what was seen in human corneal fibroblasts (Figure 6B). Upon TGF-β1 treatment, keratocan was downregulated ∼3-fold (p < 0.05), lumican remained unchanged, and decorin and perlecan both were upregulated.

The major change in response to TGF-β1 in the cord stem cell constructs may be the significant increase in sulfated GAGs. The presence of GAGs was analyzed throughout the construct using Cuprolinic Blue, which binds to sulfated moieties [24,27]. The GAG filaments were detected in either the plane of the collagen fibril (Figure 7.1B and D, “GAG” arrows) or associated with fibrils out of the plane of the image. In control constructs of both human corneal fibroblasts (Figure 7.1A) and cord stem cells (Figure 7.1C) the filaments are present and are ∼20 nm and 30 nm, respectively (Figure 7.2). Upon the addition of TGF-β1, the GAG filament length in the human corneal fibroblast (Figure 7.1B) and cord stem cell (Figure 7.1D) constructs increased significantly (p < 0.0001, Figure 7.2), indicating a similar mechanism of extracellular matrix organization.

**Figure 7:**
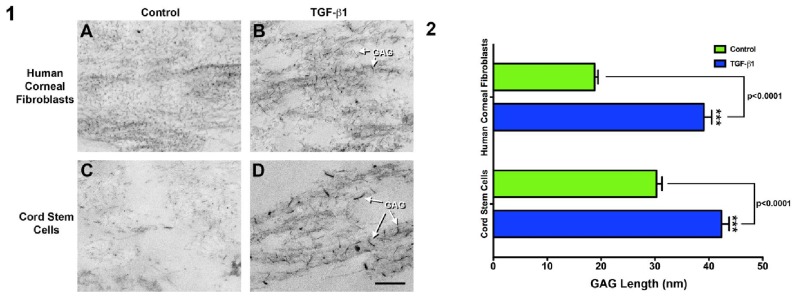
GAGs in constructs stained with cuprolinic blue (1) and length of GAG filaments quantified (2). (**1**) TEM of constructs stained with cuprolinic blue in order to visualize the GAGs in human corneal fibroblast constructs (**A**) Control and (**B**) TGF-β1 treated, and cord stem cell constructs (**C**) Control and (**D**) TGF-β1 treated. Note the increase in the GAG length with the addition of TGF-β1 to both cell lineages (B and D: “GAG” arrows). In addition, in both conditions, GAGs were present throughout the entire construct; and (**2**) Quantification of GAG fibril length in human corneal fibroblast and cord stem cell constructs. GAGs were significantly (p < 0.0001) increased in length when TGF-β1 was present in both cell lineages.

## 4. Discussion

The potential for developing cornea-like biomaterials has been greatly advanced with the use and availability of stem cells. The unique ability of stem cells to differentiate and adapt to various environments is on the verge of improving tissue integrity and stability following injury/trauma. They have been associated with repair of many organs [31,32,33], and researchers [2,3] have shown that stem cells contribute to the regeneration of a variety of tissues, such as bone, cartilage, muscle, ligament, tendon, adipose and stroma. They can be detected in various adult tissues, such as bone marrow [34], skin [35,36], retina [37], and corneal endothelium [38,39], and can now be isolated and purified using specific surface markers [40,41,42].

The present study demonstrates the ability of the cord stem cells to secrete and organize their own extracellular matrix under conditions similar to those needed by the human corneal fibroblasts [21,22,27]. The extracellular matrix produced by the cord stem cells expressed mRNA transcripts of Type I and V collagen as did the human corneal fibroblasts, and the fibril diameters were not significantly different from human corneal stromas [43]. This is a demonstration that the stem cells show the ability to differentiate and synthesize a stroma-like matrix. Healthy human corneal stroma is known to contain type I and V collagen heteromers at a ratio of 85:15, which is thought to play a major role in maintaining fibril diameters [44]. Our data suggests that cord stem cells could potentially be used as an alternative to human corneal fibroblasts, with a potentially longer life span and minimum, to no risks for the host tissue/organ. This is in agreement with recent findings by Liu and coworkers [45], where they reported that cord stem cells do not raise the immune response when transplanted into mouse corneas.

One of the most striking aspects of our study was the apparent increase in alignment of the fibrils upon TGF-β1 stimulation of the cord stem cells. The mechanisms involved in collagen fibril alignment are controversial and a more comprehensive discussion is found in the review by Ruberti and Zieske (2008) [46]. In general, several mechanisms have been postulated including template direction, cell guidance, and intrinsic properties of the collagen. Our data, which indicate that both corneal fibroblasts and cord stem cells have the ability to secrete aligned collagen, suggests that alignment is not a unique property of corneal cells. Rather, the concentration and composition of the secreted collagen and proteoglycans might direct the alignment. Our data showing the increased sulfation with TGF-β1 stimulation may be relevant to this possibility.

Cord stem cells share most of their immunophenotype with MSCs, including a cluster of differentiating markers, neural markers and extracellular adhesion molecules, as reviewed by Cao and Feng [47]. Briefly, cord stem cells were found negative for CD14, CD28, CD31, CD33, CD34, CD45, CD56, CD133, and HLA-DR, which makes them a good candidate for transplantation without raising immune responses. Their potential as biomaterials and their availability for transplantation has been exploited recently with promising results. Cord stem cells are already widely accepted as a source of hematopoietic stem and progenitor cells, and transplants have been performed for various diseases [48]. Other investigators have suggested that cord stem cells may differentiate both *in vitro* and *in vivo* into a variety of different cell lineages. Baksh *et al.* [49] assessed the potential of human umbilical cord stem cells, they found that the cord stem cells were equal or superior to the hBMSCs, showing higher proliferative potential, as well as higher capability of osteogenic, chondrogenic and adipogenic differentiation. Recently, Wang *et al.* [50] showed the benefits of using cord stem cells in a tissue-engineering model. The authors reported higher adherence of the cord stem cells to polyglycolic acid scaffolds compared to hBMSCs. More importantly they found a 2-fold increase in collagen production. In our present study, we show that these cells have at least equivalent capabilities as the human corneal fibroblasts to create an extracellular matrix that can be used as a carrier or a biomaterial in wounded corneas.

The other major structural components that are important for corneal development are the proteoglycans. These proteoglycans contain chondroitin or keratan sulfate side chains that associate with collagen fibrils, and are known to change during corneal development [51,52,53] and response to injury [18,19,54]. After injury several modifications to proteoglycans are observed, including the expression of proteoglycans with heparan sulfate side chains, and modification of other side chains, resulting in an increase in dermatan sulfate [18,19,54].

Liu *et al.* [45] successfully transplanted cord stem cells to Lum^−/−^ mice, improving corneal transparency and stromal thickness, as well as showing expression of keratocyte specific proteoglycans, such as keratocan and lumican. The study proposed that survival of these cells in mouse corneas indicated that they would also be accepted in human hosts. Our data suggest that our culture conditions containing high levels of VitC may mimic the inductive properties of the adult stroma.

Du and coworkers [55], isolated stem cells from human corneas and injected them into mice in order to investigate stromal opacity. They found that corneal transparency in the treated mice was indistinguishable from that of untreated mice, suggesting immune privilege of adult stem cells and the ability to regenerate tissue. Again, corneal specific proteoglycans were expressed, indicating their vital role to the cornea's transparency and integrity. We have previously demonstrated that the core proteins were synthesized and expressed by our 3D *in vitro* corneal fibroblasts model, and that the collagen fibrils displayed associated sulfated GAGs [27]. When the cord stem cells were placed into this model, the core proteins were also expressed. This further supports the potential of inducing these cells to produce a biomimetic cornea.

A potential problem with some of the studies listed above is that they involve the injection of the cells into a potentially compromised setting. This could be even more problematic in the case of an injury or disease where a portion of the cornea is degraded or even missing. Our studies suggest that it may be possible to grow a stroma-like tissue using cord stem cells that could be transplanted into the wound area, thus providing a scaffold for repair.

We are also intrigued by the TGF-β1-stimulated cord stem cells results, which were somewhat surprising. With human corneal fibroblasts, TGF-β1 appeared to stimulate fibrosis; however, with cord stem cells, TGF-β1 actually appeared to assemble a more aligned matrix. In addition, we found an increase in perlecan, similar to that seen in corneal fibroblasts [19,56]. While, there is no increase in the overall thickness of the construct with TGF-β1, there is a significant increase in the number of GAGs along the collagen fibrils and in the length of the GAGs, all of which are indicative of a developmental phenotype. In addition, the size of the Cuprolinic blue filaments was similar to those reported for the human corneal stroma [57]. Also, the increase in filaments along the collagen fibrils has also been reported in early reports by Cintron *et al.* [17] in wounded rabbit corneal stomas.

## 5. Conclusions

Cord stem cells have the capability of differentiating into other cell types, as well as being readily available without any host-rejection concerns. Our 3D model data shows that these cells can be stimulated to secrete a matrix similar to the one secreted by human corneal fibroblasts. Further studies are underway in order to characterize these cells *in vitro*, as well as in a cornea environment *in vivo*. Overall, cord stem cells hold great promise as a substitute for corneal transplants.
